# Development of the foregut and the formation of the trachea and esophagus in rat embryos. A symphony of confusion

**DOI:** 10.3389/fcell.2023.1092753

**Published:** 2023-02-07

**Authors:** Marco Ginzel, Nana Huber, Leopold Bauer, Dietrich Kluth, Roman Metzger

**Affiliations:** ^1^ Department of Pediatric and Adolescent Surgery, Paracelsus Medical University Hospital, Salzburg, Austria; ^2^ Department of Pediatric Surgery, University Hospital Leipzig, Leipzig, Germany

**Keywords:** foregut development, micro computed tomography, μCT, embryology, tracheal development, esophageal development

## Abstract

**Introduction:** During embryonic development, the trachea emerges from an area of the foregut, which is often referred to as “anterior” or “common” foregut tube or simply foregut. To explain this process of differentiation, four competing models exist to date. The outgrowth and watershed models propose a foregut that remains constant in length. In the outgrowth model, the trachea buds off and elongates from the foregut, while in the watershed model, a mesenchymal wedge splits the growing foregut into the trachea and esophagus. In contrast, the septation model proposes a cranial splitting and thus a shortening of the “common” foregut tube into the trachea and esophagus by an emerging septum. Finally, the splitting and extension model describes an interaction of cranial splitting of the foregut and simultaneous caudal tracheal and esophageal growth.

**Methods:** Here we examine the development of the undifferentiated foregut by micro computed tomography, which allows precise measurements.

**Results:** Our results show that this area of the foregut transforms into the larynx, a process, which is independent from tracheal and esophageal development.

**Discussion:** These observations are only consistent with the outgrowth model.

## Introduction

**FIGURE 1:**
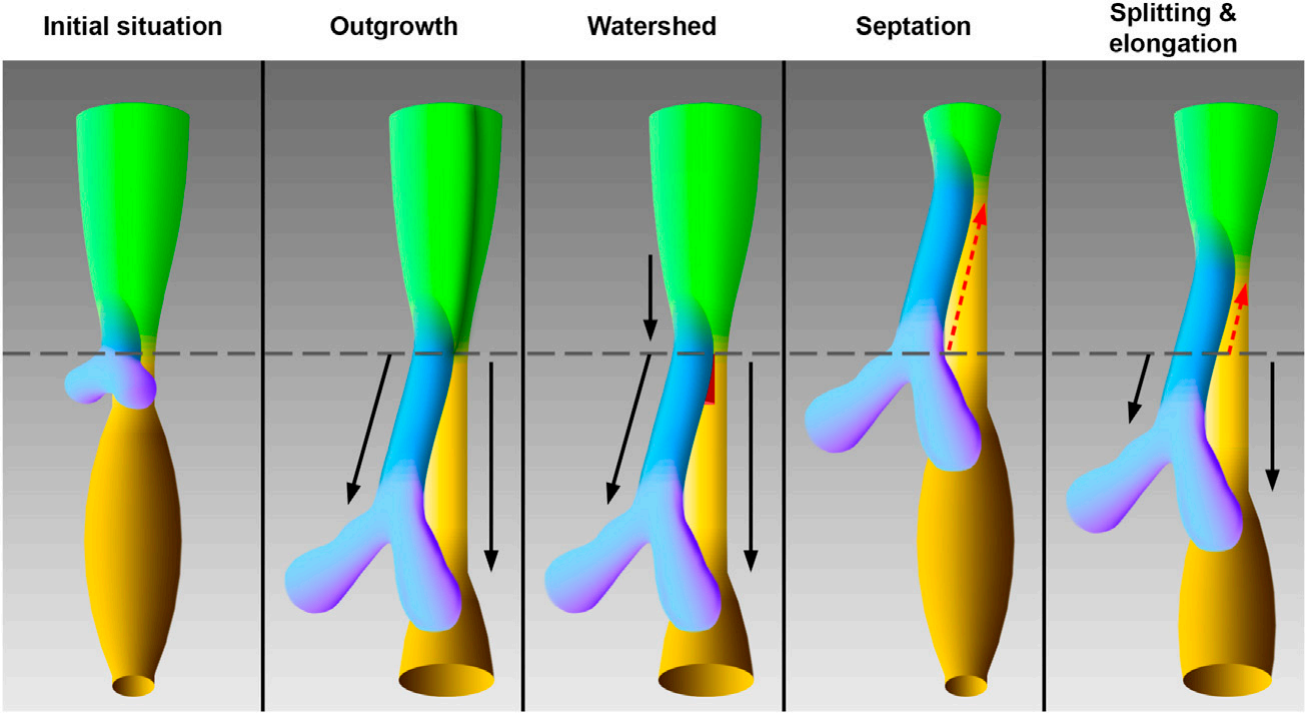
Models of tracheal development according to the original works. The initial situation of the early foregut shows lung buds without an apparent trachea, which corresponds to embryonic day 11 in rat embryos. The schematic drawings of the models correspond to embryonic day 12 in rat embryos. In the “outgrowth” model the trachea elongates from an area of the foregut, which differentiates later into the larynx (Zaw-Tun, 1982). A vertical dark green borderline indicate differentiation into larynx—hypopharynx in this foregut area. The “watershed” model postulates a mesenchymal “wedge,” which separates a descending foregut into trachea and esophagus (Sasaki et al., 2001). According to the “septation” model the common foregut is split from caudal to cranial by an evolving septum (Smith, 1957). In the “splitting and elongation” model the foregut is divided and the trachea and esophagus elongate (Que, 2015). Light green: Foregut, blue: Trachea, purple: Lung, yellow: Esophagus, ocher: Stomach, red: Mesenchymal wedge, black arrows: growth, red dotted arrows: Split, horizontal gray line: Tracheoesophageal bifurcation point.

In the foregut a region exists, which leads to the development of the pharynx, larynx, trachea, and esophagus. The present study will particularly focus on the early tracheal, esophageal and laryngeal development in the area, which was frequently called “anterior” or “common” foregut tube or just foregut in previous studies (Qi and Beasley, 2000; Sasaki et al., 2001; Ioannides et al., 2010; Que, 2015; Nasr et al., 2019). It is believed that disturbances during this differentiation process may lead to common congenital disabilities in humans, such as esophageal atresia (Merei and Hutson, 2002; Ioannides et al., 2010; Fausett and Klingensmith, 2012). Hence, the embryology of the foregut has been extensively studied. The earliest descriptions date back to 1885 and 1912 by investigating human specimens (His, 1885; Grosser, 1912; Lewis, 1912). Over the past decades, foregut development has been restudied using different animal models, such as mice, rats, chicken and *xenopus* (Qi and Beasley, 2000; Ioannides et al., 2010; Metzger et al., 2011; Que, 2015; Nasr et al., 2019). However, the respective conclusions vary to such an extent that consensus on the morphogenesis of the foregut to form the ventral trachea and dorsal esophagus has not been reached yet. Today, already four models have been described of how the trachea emerges from the foregut (Figure 1). In the so-called “outgrowth” model the trachea buds off and elongates from the foregut (Zaw-Tun, 1982). The “watershed” model describes a mesenchymal septum, serving like a wedge, which lies at the junction of the foregut and the emerging lung buds, forcing the foregut to grow separately into the trachea and esophagus (Sasaki et al., 2001). None of these models involve shortening of the foregut. In contrast, the “septation” (initially separation) model postulates a developing septum, which emerges at the lung buds and grows rostral, thereby separating the foregut into the trachea and esophagus (His, 1885; Grosser, 1912; Lewis, 1912; Smith, 1957; Ioannides et al., 2010). The latest postulated model consists of a combination of separation and thus shortening of the foregut and lengthening of the trachea and esophagus. Hence the name “splitting and extension/elongation” model (Que, 2015; Nasr et al., 2019).

Most of these models are based on histological examinations from normally developed specimens (His, 1885; Grosser, 1912; Lewis, 1912; Negus, 1924; Zaw-Tun, 1982; O'Rahilly and Muller, 1984; Nasr et al., 2019). Other methods utilized were scanning electron microscopy (Metzger et al., 2011) and even *ex-vivo* growth of a mouse esophagus (Que, 2015). In some cases, conclusions have also been drawn from studies using animal models of malformations, such as the Adriamycin esophageal atresia model (Sasaki et al., 2001; Ioannides et al., 2010). While histology provides a precise way to measure lengths within a section, creating a sagittal section from a twisted embryo is difficult. Therefore, the most common technique to determine foregut length was to create cross-sections of a defined thickness and calculate the height based on their number (Qi and Beasley, 2000; Ioannides et al., 2010). However, as we shall show, this may lead to artificial results since it is problematic to set precise landmarks, especially for histological examinations. An investigation by SEM offers detailed three-dimensional pictures of structures, which gives a better understanding of the morphology. Unfortunately, the structure of interest needs to be prepared by removing surrounding tissues, which might cause artifacts.

**FIGURE 2:**
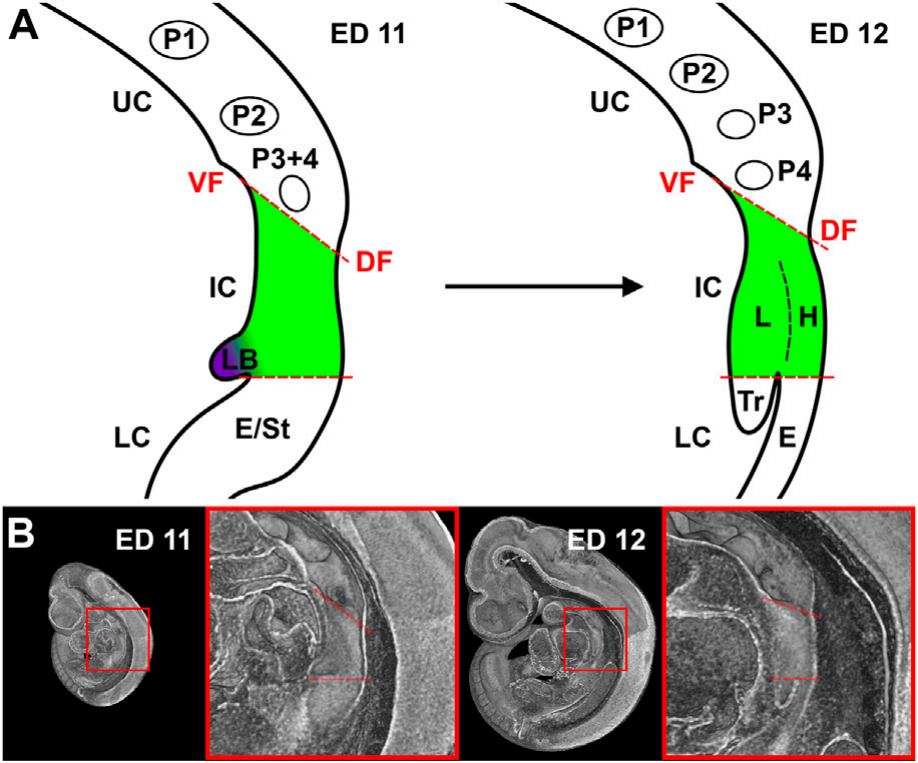
Representation of our definition of the foregut compartments. **(A)** Schematic drawing of a sagittal plane of the foregut in rat embryos at day 11 and 12. UC, Upper compartment; IC, Intermediate compartment; LC, Lower compartment; VF, Ventral fold; DF, Dorsal fold; P1-4, Pharyngeal pouches; LB, Lung bud; E, Esophagus; St, Stomach; L, larynx; H, Hypopharynx; Tr, Trachea. **(B)** Representative sagittal plane of reconstructions of rat embryos with enlarged area of interest.

Surprisingly, a great variety in the description of the examined foregut areas was observed in the cited literature, which makes it difficult for the reader and researcher to properly understand what is discussed. For practical purposes, we divided the early foregut by a borderline, the “tracheoesophageal” (TE) bifurcation point, which is characterized by Zaw Tun as the primitive pharyngeal floor. At the same time, in other studies, it is referred to “separation point,” “wedge,” “saddle” or “tracheoesophageal sulcus” (O'Rahilly and Muller, 1984; Sutliff and Hutchins, 1994; Qi and Beasley, 2000; Que, 2015). In our study, this border divides the early foregut into a lower compartment, consisting of trachea and esophagus, and an intermediate compartment, which is part of the confusion that exist. Additionally, we would define an upper compartment, consisting of the developing oral cavity and the pharynx (including the thyroid primordium among others) (Figures 2A,B). The intermediate compartment was usually defined as the area between the TE bifurcation point and the region of the 4th pharyngeal pouch, which was often referred to as “anterior” or “common” foregut tube or simply foregut (Qi and Beasley, 2000; Sasaki et al., 2001; Ioannides et al., 2010; Que, 2015). As we will show, that area can be identified as the laryngeal—hypopharyngeal (LH) primordium, which development seems to be “independent” from tracheal and esophageal growth.

Quoting a recent review; “to determine the most accurate model, quantifying changes to the lengths of the trachea, esophagus and common undivided foregut is important” (Kishimoto and Morimoto, 2021). Thus, we investigated the morphology of the developing foregut in the tracheoesophageal area in rat embryos using micro computed tomography (µCT), as this technique provides precise length and volume measurements. We used rat embryos for this study of normal embryology because several models of malformations, such as esophageal atresia, congenital diaphragmatic hernia, and anal atresia, have been established in rats, too (Thompson et al., 1978; Kluth et al., 1990; Arana et al., 2001). Thus, we have the opportunity to study these models later using µCT.

## Results

**FIGURE 3:**
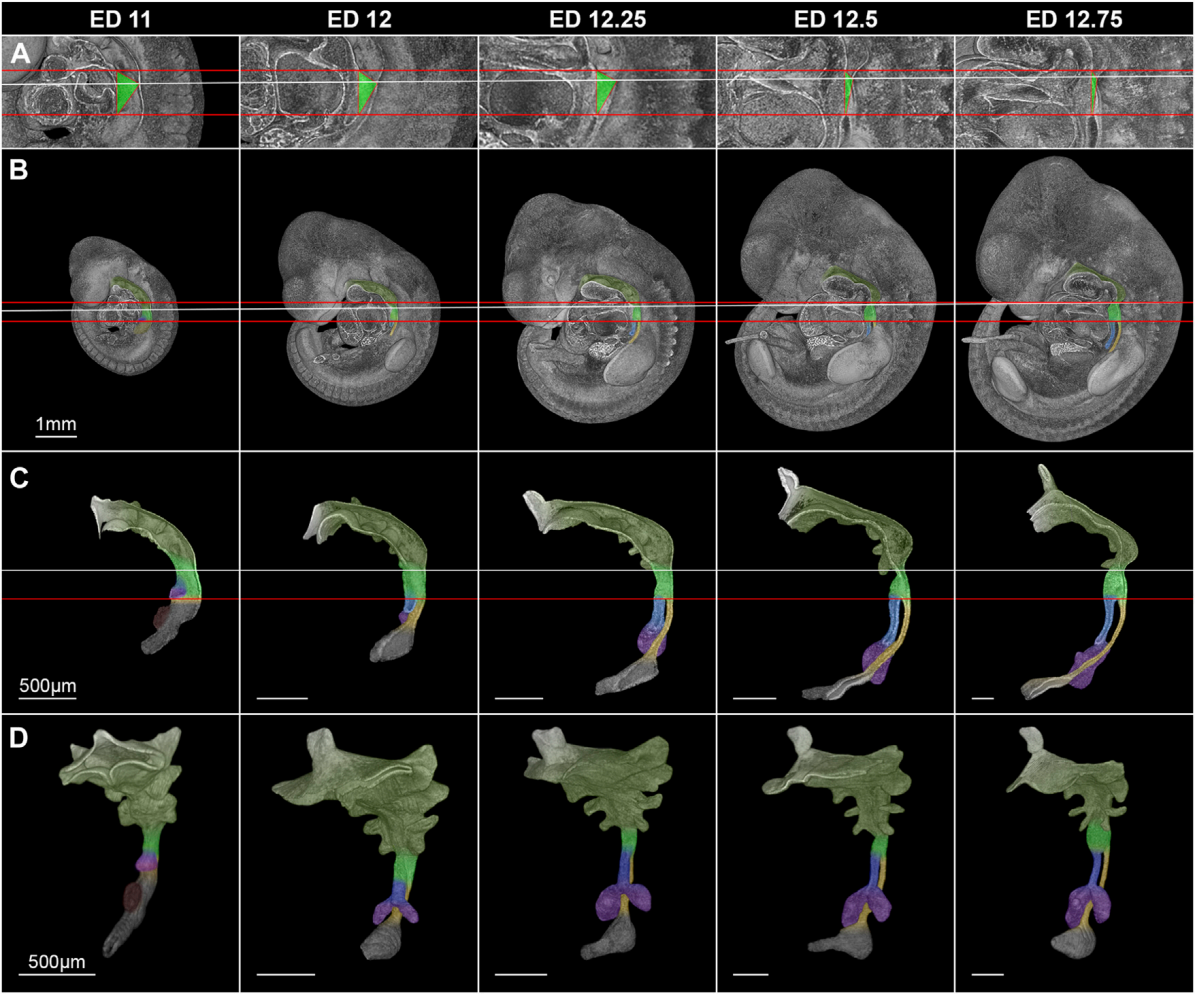
Morphological growth dynamics of the intermediate compartment of the foregut, the trachea and esophagus from ED 11 to ED 12,75. **(A)** The caudal, ventrocranial and dorsocranial measurement points for the intermediate compartment. **(B)** Rat embryos with a virtually broached foregut to scale. **(C)** Sagittal view on virtually excised foreguts including the trachea, esophagus and stomach. **(D)** Front-left view on the whole excised foreguts. The horizontal red lines indicate the height of the ventrocranial end of the intermediate compartment to the TE bifurcation point (distance ∼410 µm), while the white line shows the dorsocranial end of the TE foregut. Light green: Intermediate foregut compartment, blue: Trachea, yellow: Esophagus, purple: Lung, dark green: Upper foregut compartment (Pharyngeal foregut), dark gray **(C,D)**: Stomach.

In rat embryos, the lung buds formed at embryonic day (ED) 11 as ventral protrusions from the tracheoesophageal region of the foregut cranial of and in direct contact with the developing stomach (Figure 3). This fold caused by the lung buds marks the TE bifurcation and thus the starting point of tracheal development. 130 µm caudally of the lung bud the liver primordium formed another ventral bud from the developing stomach (Figure 3). At ED 12, a small portion of the trachea and esophagus has developed, and the liver primordium has differentiated into liver. In rats, it takes only one more day to form a distinct portion of the trachea and esophagus. Thus, we took samples every quarter day for a better resolution, referring to it as ED 12.25, ED 12.5 and ED 12.75. We analyzed the morphology and morphometry at these time points of the intermediate foregut compartment and the trachea and esophagus.

**FIGURE 4:**
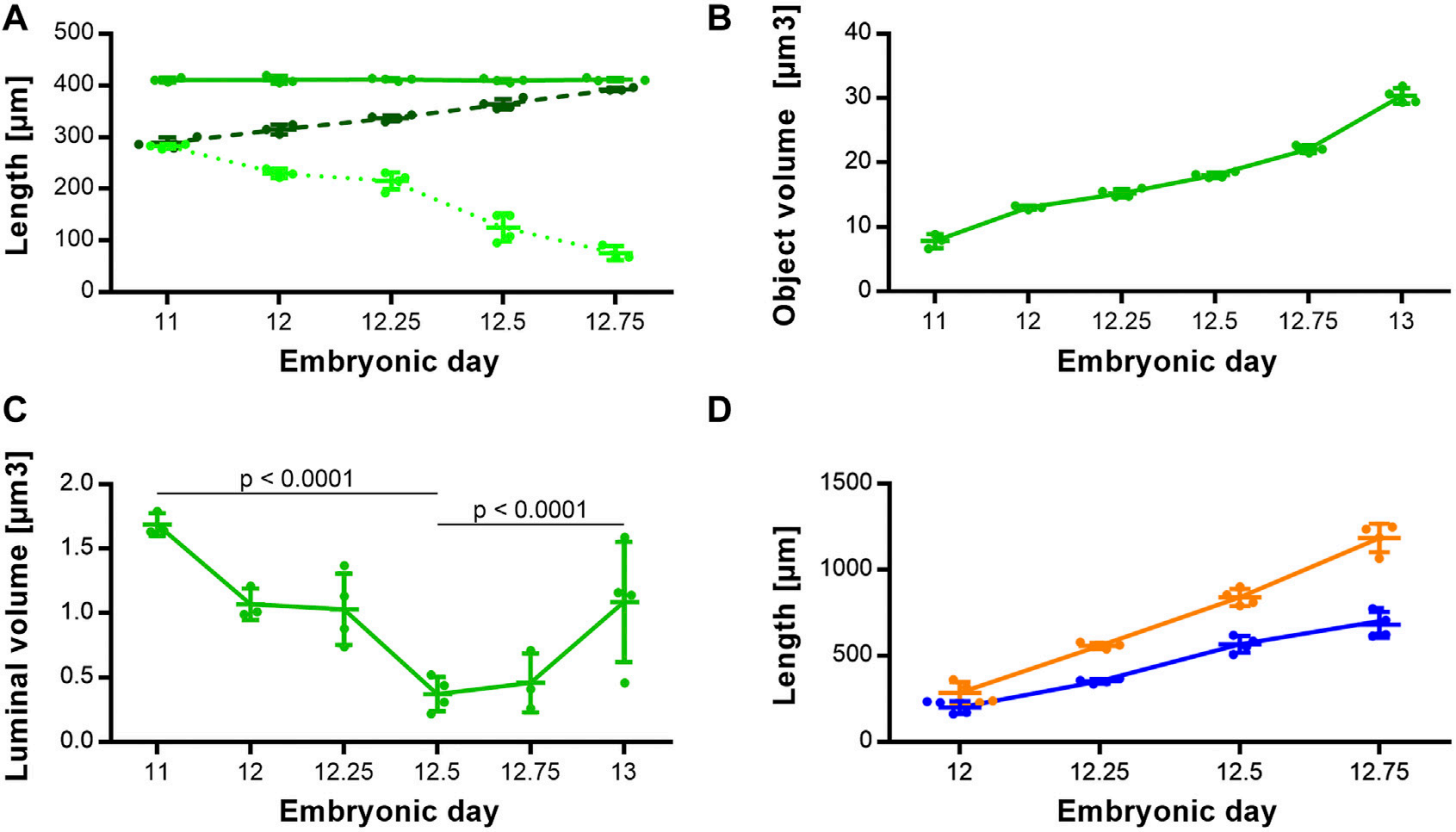
Morphometrical growth dynamics of the intermediate compartment, trachea and esophagus. **(A)** Length measurements of the intermediate compartment of the foregut (Solid green line: TE bifurcation to the most ventrocranial point; *p* = 0.9757. Dashed dark green line: TE bifurcation to the most dorsocranial point; *p* < 0.0001. Dottet light green line: Distance from the most ventrocranial to the most dorsocranial point; *p* < 0.0001). **(B)** Object volumes of the intermediate compartment of the foregut; *p* < 0.0001. **(C)** Luminal volumes of the intermediate compartment of the foregut. **(D)** Length measurements of the trachea (blue line) and esophagus (orange line); both *p* < 0.0001.

Morphologically, the TE bifurcation point defined the caudal border of this intermediate compartment. Additionally, we found a cranial border, which can be identified by a dorsal and ventral epithelial fold, connected by lateral folds. The dorsal fold was located in the area between the 3rd and 4th somites, the ventral fold approximately at the area of the 4th pharyngeal pouch (Supplementary Figures S1–S5). From ED 11 to ED 12.75, these folds converged by a dorsal movement of the ventrocranial fold (Figures 3A, B), thereby narrowing the foregut in this region and forming the cranial entrance from the pharynx into the developing larynx—hypopharynx (Figures 3C, D). The dorsal wall of the pharynx also folded into dorsal direction in this period (Figure 3). At around ED 12.5 the differentiation of the intermediate compartment of the foregut into the larynx (ventral) and hypopharynx (dorsal) was visible by lateral mesenchymal thickening, which continued over time. Our measurements supported these observations. The most ventrocranial point of the intermediate compartment remained at a constant distance of 410,5 µm (±3.8 µm) to the TE bifurcation point from ED 11 to ED 12.75, but moved in dorsal direction, thereby narrowing the pharynx—larynx transition zone from 281.8 µm (±4.8 µm) to 74.9 µm (±13.6 µm). The most dorsocranial point of the intermediate compartment increased its length linear from 282.5 µm (±11.9 µm) at ED 11–369.1 µm (±20.1 µm) at ED 12.75 (Figure 4A). The object volume increased linear from 7.8 µm (±1.1 µm) to 22.1 µm (±0.6 µm) (Figure 4B). However, the luminal volume (total volume minus object volume) decreased from 1.69 µm (±1.1 µm) at ED 11 to 0.37 µm (±0.13 µm) at ED12.5 and increased after that to 1.09 µm (±0.47 µm) at ED 13 (Figure 4C).

The trachea increased its length from 199.3 µm (±37.8 µm) at ED 11–680.4 µm (±75.1 µm) at ED 12.75 and the esophagus from 285.8 µm (±63.1 µm) to 1185 µm (±82.8 µm) respectively, while both growth curves are linear (Figure 4D).

A mesenchymal wedge at the site of the initial lung bud fold could not be observed. Although cells are present at this location, they do not form a solid structure but rather soft tissue, in contrast to the well-defined trachea and esophagus (Supplementary Figures S1–S5; Supplemental Videos S1–S5).

**FIGURE 5:**
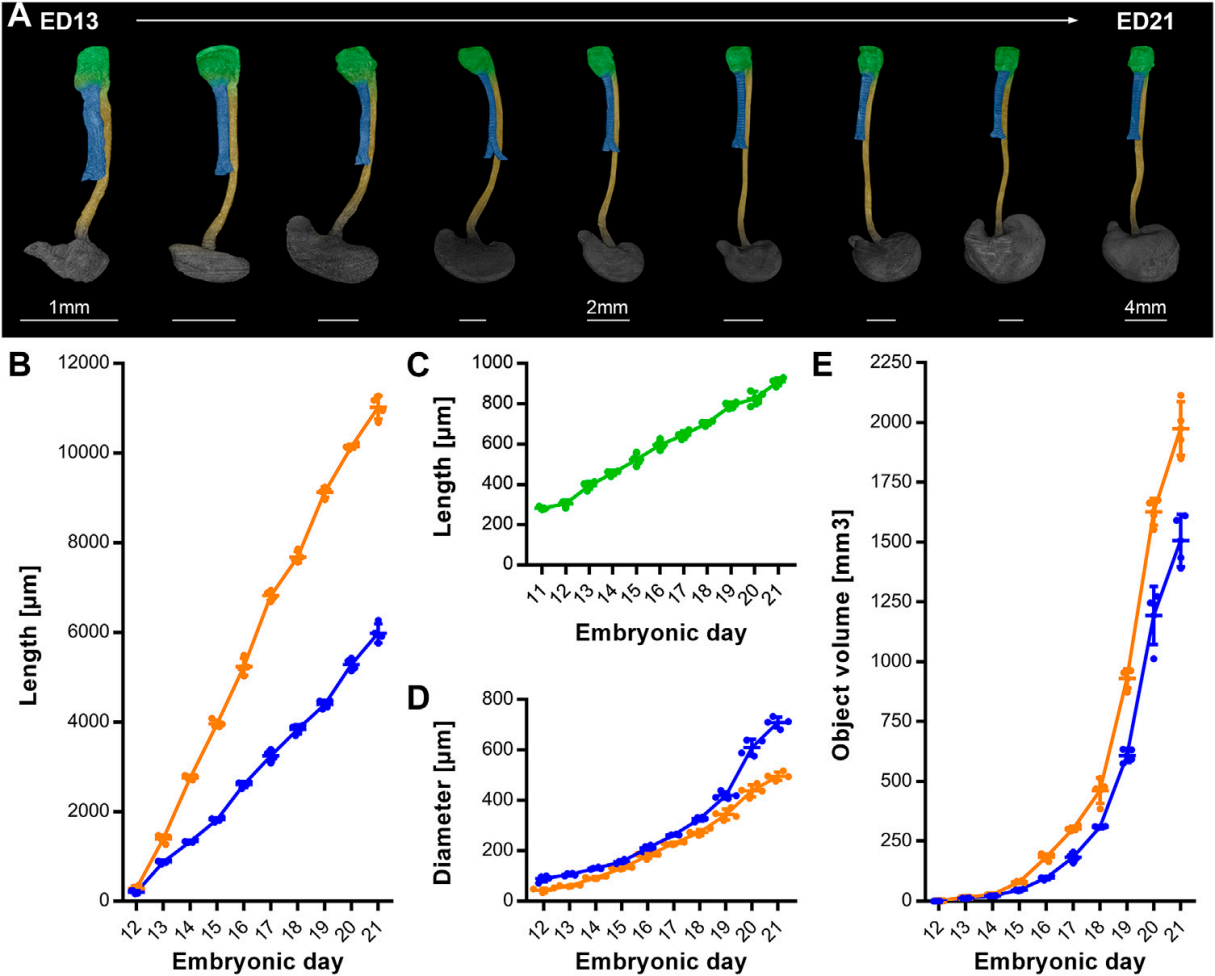
Growth dynamics of the intermediate compartment/ larynx, trachea and esophagus from ED11 to ED21. **(A)** Representative reconstructions showing the growth of the larynx, trachea, esophagus and stomach over time; *p* < 0.0001. Light green: Larynx - hypopharynx, blue: Trachea, yellow: Esophagus, dark gray: Stomach, **(B)** Measured lengths of trachea (blue line) and esophagus (orange line) over time; *p* < 0.0001. **(C)** Measured lengths of the larynx (light green line) over time; *p* < 0.0001. **(D)** Measured diameter of trachea (blue line) and esophagus (orange line) over time; *p* < 0.0001. **(E)** Measured object volumes of trachea (blue line) and esophagus (orange line) over time; *p* < 0.0001.

To complete the analysis over time, we measured the growth patterns until ED 21, 1-day prior to birth (Figure 5A). From ED 11 to ED 21 the intermediate compartment (later larynx/hypopharynx) length continuously triples from around 350 μm–910.5 µm (±18.7 µm) (Figure 5C). The trachea increased its length linear until ED 21 from 205.3 µm (±66.7 µm) to 5979.8 µm (±213.3 µm) (Figure 5B), while its diameter exponentially increased from 88.4 µm (±13 µm) to 709 µm (±21.4 µm) (Figure 5D). From ED 12 to ED 21 the esophagus length increased linearly from 285.8 µm (±63.1 µm) to 11,019.8 µm (±260.4 µm) (Figure 5B) and the diameter from 43.1 µm (±8.4 µm) to 496 µm (±16.8 µm) (Figure 5D). The growth dynamics of the esophageal diameter could be linear or slightly exponential. The tracheal and esophageal object volumes increased exponentially from ED 12 to ED 20, from 0.7 (±0.06 µm) to 1167.87 (±133.28 µm) and 1.21 (±0.08 µm) to 1614.87 (±61.15 µm) respectively, roughly doubling every day, while from ED 20 to ED 21, their growth slows down (Figure 5E).

Of note, the measured distances and volumes are not meant to be understood as absolute values, as the critical point drying process comes with a shrinkage of the whole embryo. Therefore, the measured lengths of fresh and wet samples may differ, but the proportions of samples of different ages are preserved and consistent.

## Discussion

In the earliest descriptions of the developing foregut from 1885 and 1912, the authors believed that the trachea develops by separation of a “common” foregut. Here, a mesenchymal fold formed by the lung bud ascends cranially, dividing the foregut into the trachea and esophagus (His, 1885; Grosser, 1912; Lewis, 1912). Around 50 years later, Smith modified this theory, proposing that an emerging endodermal septum instead induces the separation of the foregut rather than an ascending notch. He wrote; “Ridges of endodermal cells develop from the lateral walls at the caudal end of the lung bud region of the foregut. The union of these proliferating ridges, within the lumen of the foregut, divides it into a ventral respiratory and a dorsal digestive portion” (Smith, 1957). This is, in short, how the “septation model” has come into existence. Although it has since been questioned and partly refuted by several publications (Zaw-Tun, 1982; Sutliff and Hutchins, 1994; Metzger et al., 2011), it is still viable in textbooks and finds attention (Ioannides et al., 2010; Que, 2015; Nasr et al., 2019), which may have biased investigators.

To emphasize this, the first investigations were carried out in human specimens. Usually, a maximum of 1-2 specimens have been investigated per age group, as those samples were and are still rare. Some of the first drawings by Wilhelm His from 1885 suggest a likewise constant length or in his perspective a shrinkage of the foregut by “the separation of the respiratory tube [,which] progresses from below upwards” (translation of the original German text by google translate (His, 1885)), which probably was the case, considering the variance in human specimens. For instance, it is reported, that the size of a human embryo ranges from 5 to 7 mm at Carnegie stage 14 and 7–9 mm at stage 15 respectively (Human Embryology, 2022). These initial pictures and reports might have led to a persistent belief, that the foregut either remains constant in length or shrinks, which is reflected in the proposed models.

More recent studies have established another modified version of the septation model by adding a simultaneous extension of the trachea and esophagus, thereby combining separation and growth. In 2015 Que “[…] shows a rostral translocation of a saddle-like structure that splits the anterior foregut into the trachea and esophagus” obtained by *ex vivo* “live-imaging of actively separating E (D) 9.5 […] (mouse) foregut […]. Meanwhile, the extension of the trachea and esophagus is observed as the saddle-like structure moves up, suggesting a co-existence of splitting and extension” (Que, 2015). Although this is a remarkable technique, an excised foregut from an ED 9.5 mouse embryo cultivated *ex vivo* cannot be an accurate method to determine lengths precisely. As all connections to other tissues and the embryo have been removed, the shape of the growing foregut looks quite deformed as seen by the thick and short esophagus (Que, 2015). The author admits that it might have been an artificial observation. Additionally, a quantification is missing. Thus, an “n of one” is questionable to support a new model. But, support came, in 2019, from another study. Here, the authors examined foregut development in *xenopus* and mice by histology which supported the assumption of splitting and elongation (Nasr et al., 2019). However, most of the proposed models are not only descriptions of normal embryology, but ideally also aim to explain congenital malformations such as esophageal atresia (Smith, 1957; O'Rahilly and Muller, 1984; Sasaki et al., 2001; Ioannides et al., 2010). This aim may have indirectly caused an additional bias on research of normal embryology, as it is generally believed, that there is a specific breaking point between normal and abnormal development, which is essential to identify. In this context, it is interesting to note, that in most models of tracheoesophageal development the formation of the larynx is rarely mentioned compared to the extensively discussed formation of the TE bifurcation point. Only O’Rahilly and Zaw-Tun addressed this differentiation process (Zaw-Tun, 1982; O'Rahilly and Muller, 1984). Zaw-Tun mentioned the area of the intermediate compartment of the foregut as “laryngeal sulcus” and simultaneously questioned the theory of a splitting foregut. He pointed out that the TE bifurcation point stays at a constant level, refuting a shortening of the foregut through an upwards movement of a septum (Zaw-Tun, 1982). Even O’Rahilly, whose former studies strongly supported the septation model (O'Rahilly and Boyden, 1973; O'Rahilly and Tucker, 1973; O'Rahilly, 1978) stated in 1984 that the TE bifurcation point indeed stayed at the same level. However, he reinterpreted the “septation ridges” (which should divide the foregut into trachea and esophagus according to the septation model) into a mesenchymal septum underneath the TE bifurcation point (O'Rahilly and Muller, 1984). Later, Sasaki et al. described a “mesenchymal portion with condensed cellularity” below the TE bifurcation point (Sasaki et al., 2001). They hypothesized that this structure might serve as a wedge, leading to the watershed model, which we will discuss later. In 1994, Sutliff et al. showed that the TE bifurcation point remained at a constant level between the 5th and 6th somite, consistent with our results (Sutliff and Hutchins, 1994). Therefore, the TE bifurcation point is a rational landmark for the boundary between the caudal end of the intermediate compartment and the lower tracheal and esophageal compartment (Zaw-Tun, 1982; O'Rahilly and Muller, 1984; Sutliff and Hutchins, 1994; Qi and Beasley, 2000).

Other studies used staining of the thyroid transcription factor 1, also known as NK2 homeobox 1 (Nkx2.1) to focus on the respiratory development at the TE bifurcation point, concluding that the absolute length of the stained foregut region shrinks (Ioannides et al., 2010; Nasr et al., 2019). However, they had not demonstrated that the cranial endpoints marked by Nkx2.1 (which do not represent the cranial endpoints of the intermediate compartment) were static. Yet, this would be a prerequisite for their theory. Moreover, the authors had different interpretations of the morphology behind the staining. Ioannides stated that “the respiratory foregut, between the subglottic larynx and the tracheal bifurcation, was identified by immunohistochemistry for Nkx2.1”(Ioannides et al., 2010). In contrast Nasr limited the positive staining to the “tracheal epithelium” only (Nasr et al., 2019). Comparing our morphological data with the staining images of these studies, we believe that the Nkx2.1 staining also includes parts of the laryngeal primordium and progressive differentiation into the larynx might lead to a decrease of Nkx2.1 positive cells in this developing region.

For the cranial end of the intermediate compartment of the foregut, e.g., the 4th pharyngeal pouch was used in previous studies (Qi and Beasley, 2000). In our study, we morphologically identified two landmarks from ED 11 to ED 12.75 (the dorsal and ventral epithelial fold), which we measured. We found that the ventral length differs from the dorsal length. The distance between the TE bifurcation point and the ventrocranial endpoint remained constant from ED 11 to ED 12.75, while the distance between the TE bifurcation point and the dorsocranial endpoint was increasing linear in this period. In addition, we measured the distance between these two cranial endpoints and found that the distance shortens over time by a dorsal movement of the ventrocranial endpoint, thereby forming the cranial entrance into the developing larynx—hypopharynx (Figures 3, 4). This means, that a shorting of the distance between the TE bifurcation point and the cranial endpoints of the intermediate compartment does not take place, refuting the ideas of a complete or partial splitting or dividing of a “common” foregut tube.

Our volumetric measurements of the intermediate compartment showed an increasing object volume over time, indicating tissue growth in this area, while the lumen in that region is decreasing until ED 12.5. Thereafter, the lumen increased again. Morphologically, these changes were caused by mesenchymal tissue growth ventral and lateral of the intermediate foregut epithelium, thus reducing the lumen of this area to a small slit-like structure. The result of this process of differentiation is the formation of the early larynx—hypopharynx. In previous studies, measurements of the developing fetal larynx showed a 2-3 fold increase in height until birth, depending on the time interval (Harjeet et al., 2010; Liberty et al., 2013). Our results are in accordance to these observations (Figure 4).

This leads us to the first main conclusion. The intermediate compartment of the foregut that we have defined, which was formerly called the “anterior” or “common” foregut tube, is not the area where the trachea and esophagus are divided by any process of separation or septation, but rather the area of the primordium of the early larynx—hypopharynx, as Zaw Tun has already stated. The trachea and esophagus grow independent from the processes in this intermediate compartment.

This leaves us with the outgrowth (Zaw-Tun, 1982) and the watershed model (Sasaki et al., 2001). The main difference between these models is the existence of a mesenchymal septum in the watershed model combined with different growth dynamics. While in the outgrowth model the lengthening of the trachea and esophagus occurs only caudal to the TE bifurcation point, in the watershed model growth takes place primarily proximal and possibly distal to the TE bifurcation point (Sasaki et al., 2001). This model uses the idea of mechanically separating an undivided part of the foregut into the esophagus and trachea by this mesenchymal wedge (Sasaki et al., 2001). However, in our study we could not detect a structure that might serve as a wedge (Supplementary Figures S1–S5; Supplemental Videos S1–S5). The first dense structure we could detect emerging at the TE bifurcation point was the beginning of cartilagation at ED 15, which also appeared at the ventrocranial part of the larynx and the trachea itself. However, at ED 15, tracheal development has already taken place. Therefore, the theory of foregut differentiation caused by a mesenchymal wedge appears somewhat artificial with the aim to explain the pathogenesis of esophageal atresia.

Overall, our results support the outgrowth model, or to be precise, most of Zaw-Tun’s work (Zaw-Tun, 1982), with the modification of a transforming LH primordium which lengthens at its dorsocranial end. We are aware that this contrasts with the majority of former studies investigating tracheal and foregut development (His, 1885; Grosser, 1912; Lewis, 1912; Smith, 1957; O'Rahilly and Muller, 1984; Sutliff and Hutchins, 1994; Sasaki et al., 2001; Ioannides et al., 2010; Que, 2015; Nasr et al., 2019). However, previous discussions about the validity of the outgrowth model include some incomprehensible arguments and conclusions, from the demand for rapid tracheal growth (Ioannides et al., 2010; Que, 2015) or that the failure of tracheal growth is likely to be the primary cause of esophageal atresia and TE fistula according to the outgrowth model (Ioannides et al., 2010). This does not appear to be necessary as we do not see a period of rapid tracheal growth but its formation occurs non-etheless.

However, we can only measure lengths and volumes making it hard to distinguish between push and stretching. For instance, the trachea of the *ex vivo* cultivated mouse foregut showed relatively normal morphology. In contrast, the thickened and shortened shape of the esophagus from the study of Que et al. indicates that it might be under tension inside the organism (Que, 2015), as our datasets show a slim esophagus (Ø ≈ 45 µm) in rat embryos of comparable age. In 1982, Zaw-Tun pointed out that the increasing distance between the respiratory primordium and hepatic primordium is likely induced by the growth of the heart by geometrical progression, which also contributes to a stretch of the early foregut (Zaw-Tun, 1982). We cannot rule out that tension and pressure induced by other organs, such as the heart and the developing liver, might play a role in foregut development. However, our data show a linear increase in length of the trachea and esophagus with an exponential increase in volume, indicating no active stretching under physiological conditions (Figures 4, 5).

This brings us to the second point that, according to the outgrowth model, the failure of tracheal growth should be the primary cause of esophageal atresia and TE fistula. We believe that this conclusion is highly speculative as observations in cases of tracheal and/or esophageal malformations show (Kluth, 1976). The trachea seems to be a stable structure, which is usually formed normally or with minor malformations in cases of esophageal atresia, diaphragmatic hernia, Gastroschisis, or other malformations. Only the esophageal part may be interrupted or malformed in children with esophageal atresia and usually (87%) forms a fistula at the height of the bronchial bifurcation point (Spitz, 2007). Moreover, if the assumption of a tracheal growth deficiency is valid, observed cases of tracheal atresia or agenesis must have been combined by extreme esophageal maldevelopment. In contrast to this, in cases of tracheal atresia or agenesis, the esophagus is not absent, but serves as a substitute to the malformed or absent trachea. This means that a failure of tracheal growth is unlikely to be the cause for esophageal atresia, which was also not concluded by Zaw-Tun, but rather stated by Ioannides et al. (Ioannides et al., 2010). Further investigations of embryos with esophageal atresia are needed to answer its pathogenesis without making too many assumptions. Our work only aims to describe normal foregut development with morphological and morphometric techniques. As the morphology of rat embryos at the appropriate ages of tracheal development is similar to human embryos (Blechschmidt, 1963; Steding, 2009) and our results are consistent with those of Zaw Tun who investigated human embryos (Zaw-Tun, 1982), we hypothesize that the differentiation processes described here could also occur in humans.

## Material and methods

### Ethics and animal housing

Animal care and experimental procedures were approved by the institutional review board (state directorate Saxony, Referat 25, veterinary and food monitoring, Braustraße 2, 04,107 Leipzig. Proposals: T14/15, T44/16, T13/18). This study neither involve wild animals nor samples collected from the field. Animals were housed at the Medical Experimental Center of the University of Leipzig in rooms with a controlled temperature (22°C), humidity (55%), and 12 h light–dark cycle. Food and water were supplied ad libitum.

### Sample preparation

The sample preparation and subsequent scanning process have been described previously (Ginzel et al., 2021). In brief, Sprague-Dawley rats were mated, and pregnancy was verified by the presence of a vaginal smear. Staging was performed by definition of the gestational age, with the day of positive vaginal smear defined as embryonic day 0 (ED 0). Pregnant rats were euthanized by an overdose of pentobarbital [300 mg/kg BW], and embryos were harvested. Overall, 53 embryos, regardless of their sex, aged from ED11 to ED21 were analyzed for the current study. Mostly four embryos per age group, with the exception for ED11, ED12 and ED12.75 (n = 3) were analyzed. The embryos were fixed in Bouin’s solution at RT for 3 days and afterwards stored in 80% ethanol. To enhance image contrast, complete embryos were dehydrated with the “critical point” drying technique utilizing the critical point dryer CPD 2 (Pelco, Ted Pella, Inc., CA, United States).

### Micro-CT scanning

Each rat embryo was analyzed using SkyScan 1172-100-50 (Bruker microCT, Kontich, Belgium). All samples were scanned with 40 kV and 250 μA without filter. The voxel size ranged from 2.04 to 7.63 μm, depending on the specimen size. Images were reconstructed with the scanner software (NRecon 1.7.0.4; Bruker) and converted to a bitmap-file-format.

### Segmentation

The segmentation of embryonic structures was performed by CTAnalyzer (CTAn®, Version 1.16.1.0; Bruker). The structures were manually segmented by generating a series of regions of interest (ROIs) around the embryonic structure to extract the information.

### Used landmarks of investigated structures

We analyzed the morphology and measured the lengths and volumes of the developing trachea, esophagus and the intermediate compartment of the foregut. The lengths and volumes of the developing trachea was measured from the TE bifurcation point to the bronchial bifurcation point. The lengths and volumes of the esophagus was measured from the TE bifurcation point to the area of the later emerging diaphragm. For the intermediate compartment, we measured the distances from the TE bifurcation point to the most ventrocranial and most dorsocranial point, and the distance between these points (Figure 2A, Supplementary Figures S1–S5). The alignment of the embryos for measurement are illustrated in the Supplementary Figures S6–S10. Additionally, we measured the object volumes of the developing intermediate compartment. From ED13 onwards, we measured the respective length of the larynx/hypopharynx region from the TE bifurcation point to the pharynx transition zone to complete the measurements over time.

### Statistics and reproducibility

After data segmentation (CT Analyzer, Bruker microCT), the 3D viewing software CTvox® (Bruker microCT) was used to produce volume rendering and virtual sections for graphical illustrations and videos. Results are expressed as single data points ± standard deviation (SD). For comparison of lengths and volumes over time, one-way analysis of variance (ANOVA) with Bonferroni *post hoc* tests was used. Graphs were designed with GraphPad Prism (La Jolla, CA, United States), *p*-values were calculated with the software SPSS (Version 26, IBM®, Armonk, NY, United States) and considered significant when <0.05. The datasets used in this study are openly available under Publissio ZB MED Information Centre of Life Science at https://doi.org/10.4126/FRL01-006424446.

## Data Availability

The datasets presented in this study can be found in online repositories. The names of the repository/repositories and accession number (s) can be found below: https://doi.org/10.4126/FRL01-006424446.
