# Supplementary figures and images for: Development of the foregut and the formation of the trachea and esophagus in rat embryos. A symphony of confusion
Source: Front Cell Dev Biol. 2023 Feb 7;11:1092753. doi: 10.3389/fcell.2023.1092753 (PMC9941168; doi:10.3389/fcell.2023.1092753)

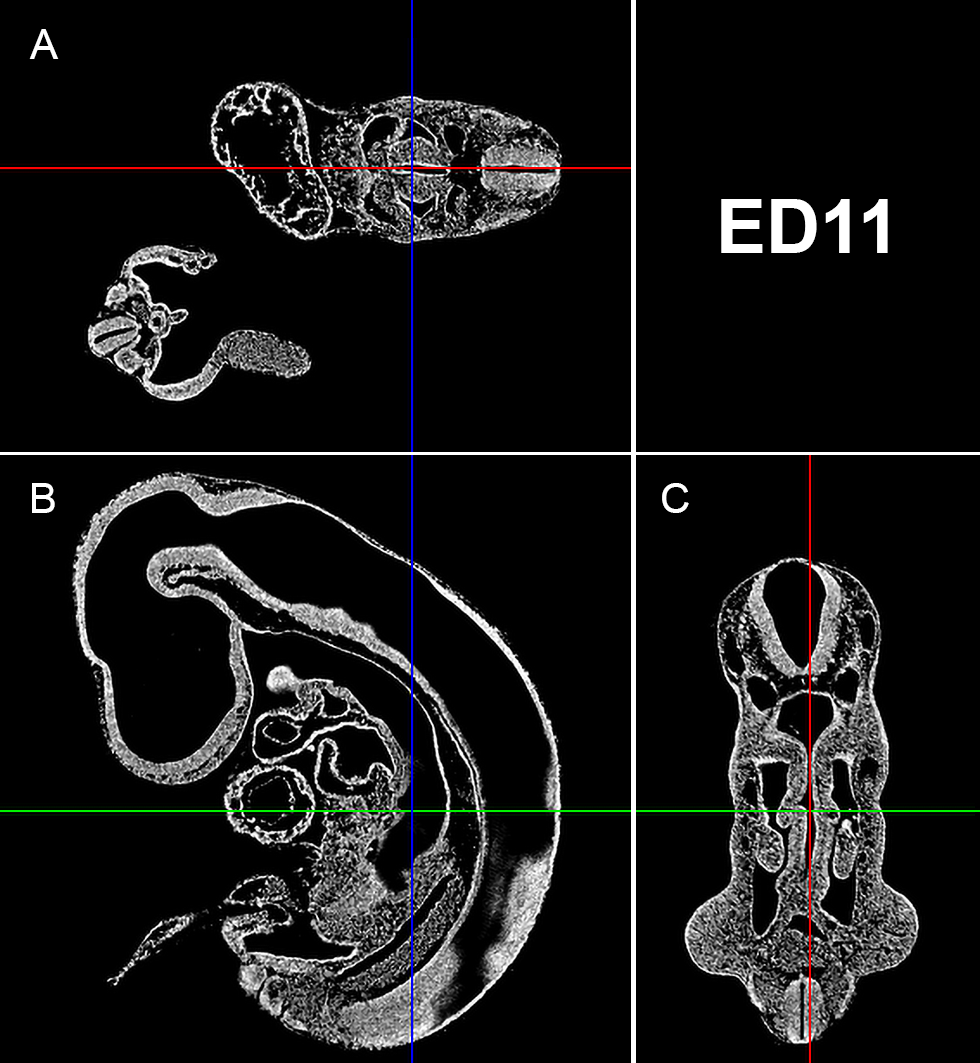

Supplement: Supplementary file 1 [file Image6.TIF]

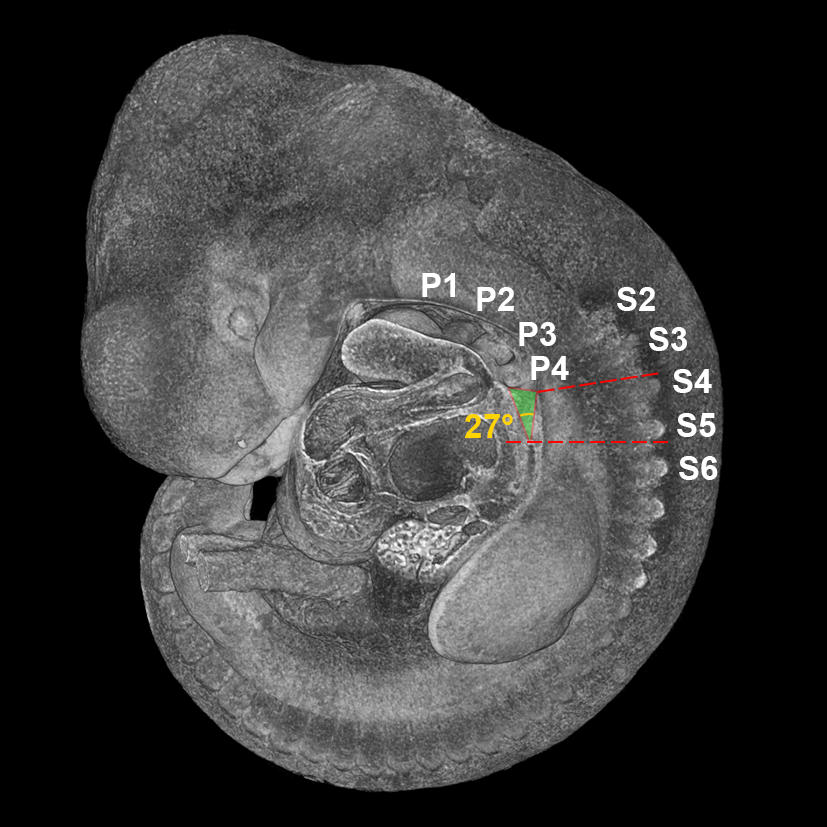

Supplement: Supplementary file 3 [file Image3.TIF]

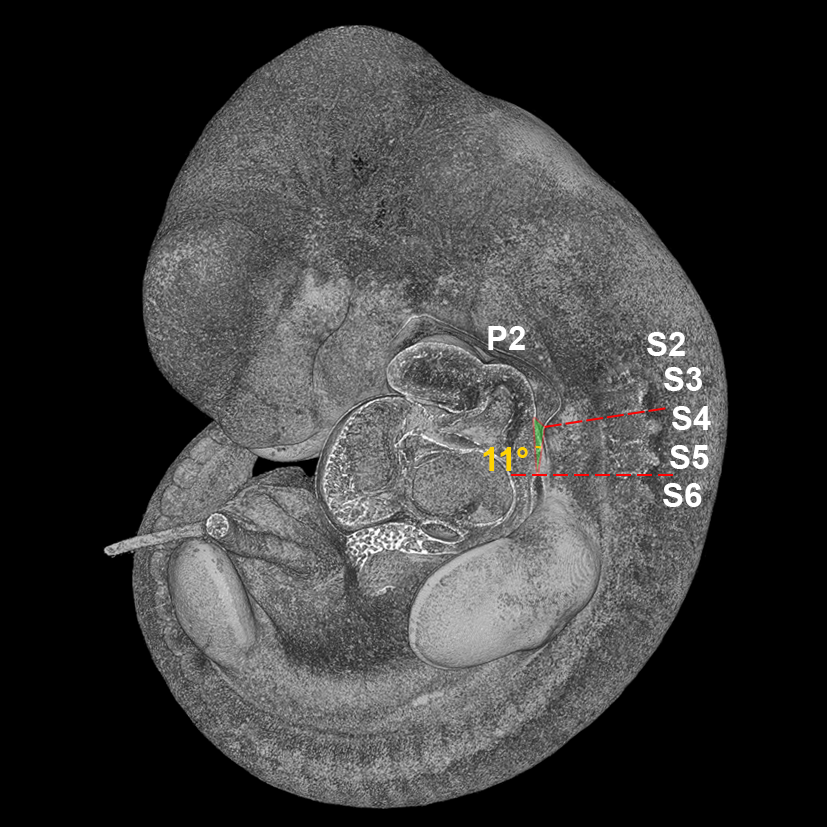

Supplement: Supplementary file 4 [file Image4.TIF]

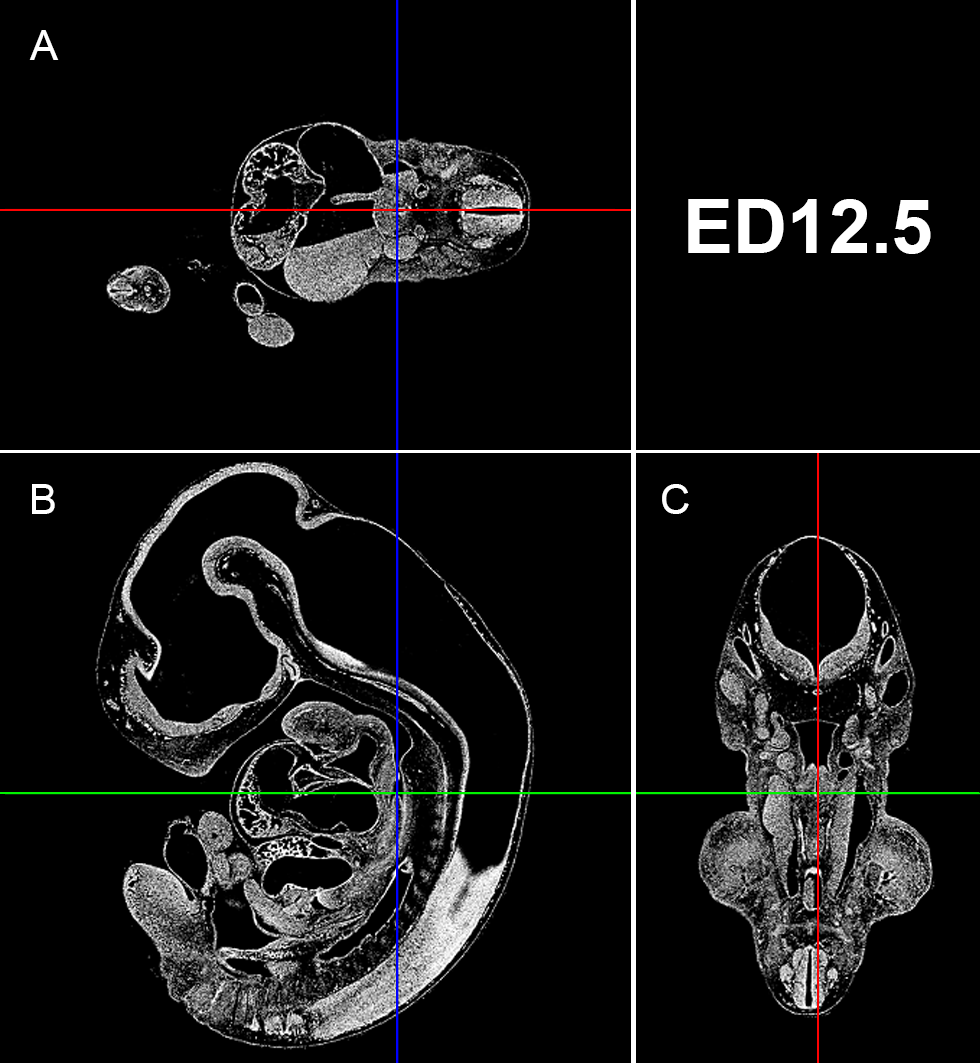

Supplement: Supplementary file 5 [file Image9.TIF]

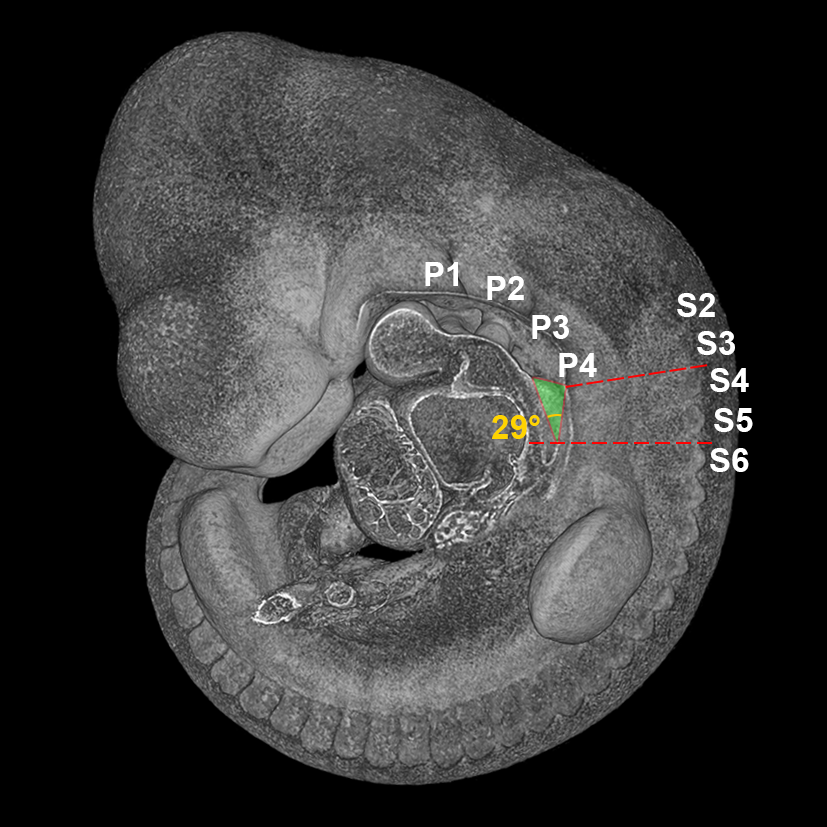

Supplement: Supplementary file 6 [file Image2.TIF]

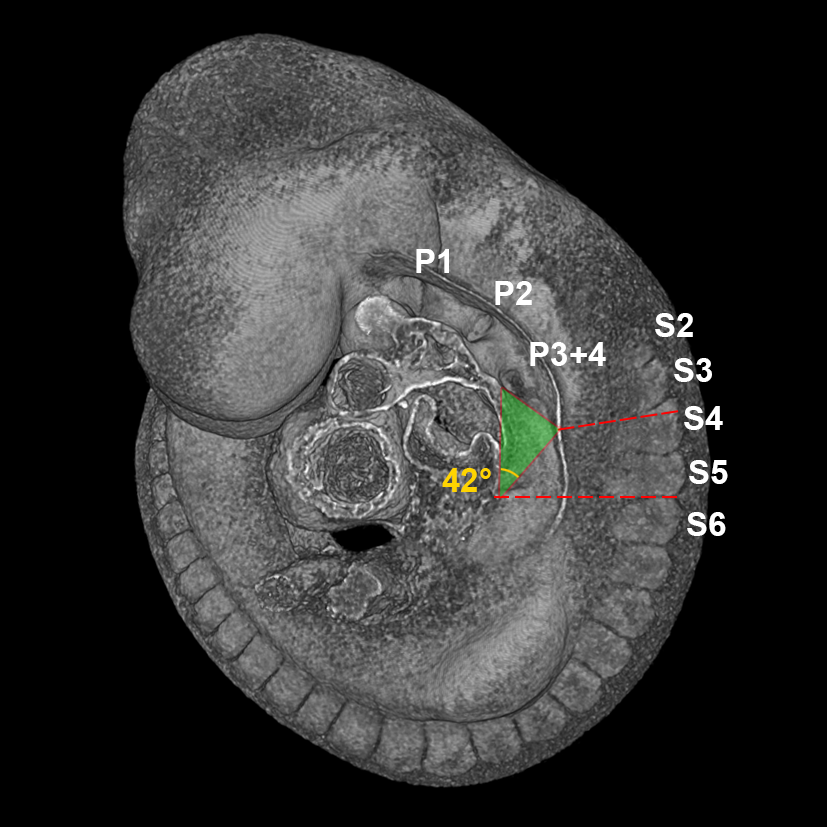

Supplement: Supplementary file 7 [file Image1.TIF]

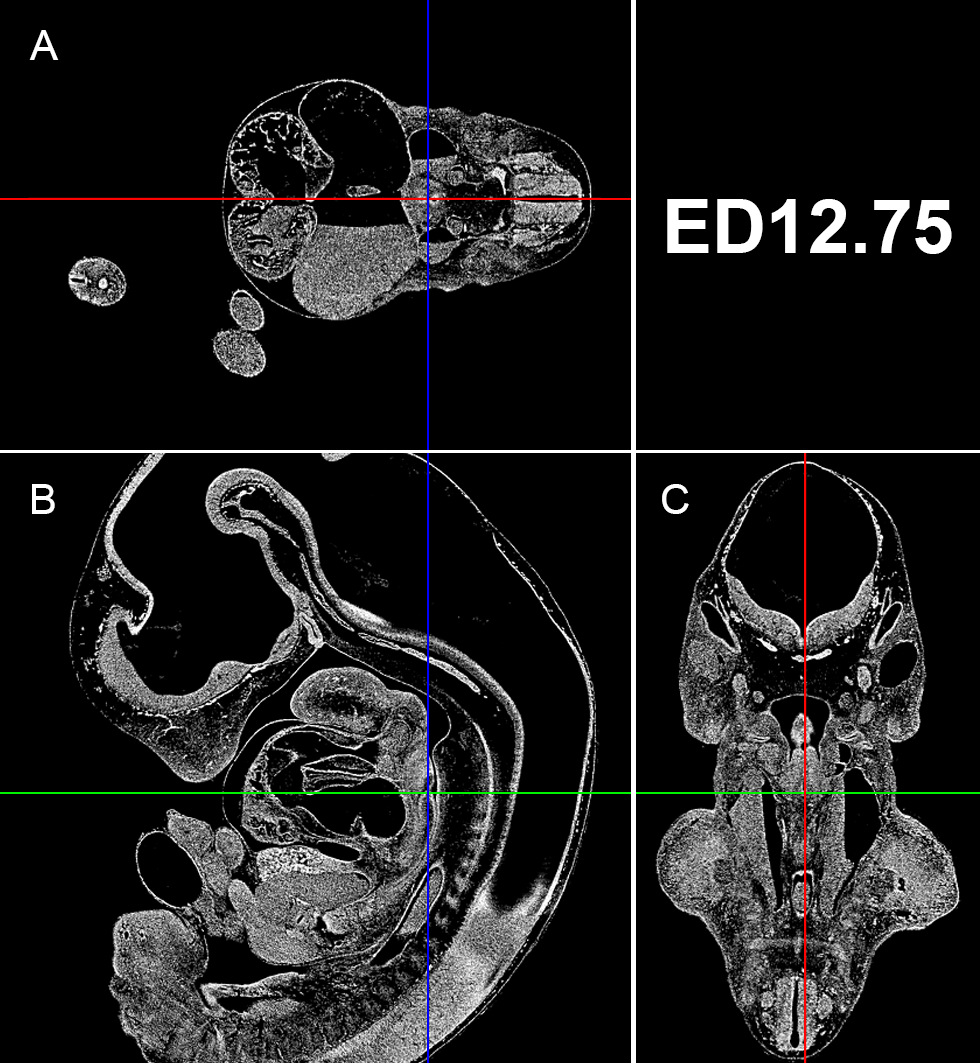

Supplement: Supplementary file 8 [file Image10.TIF]

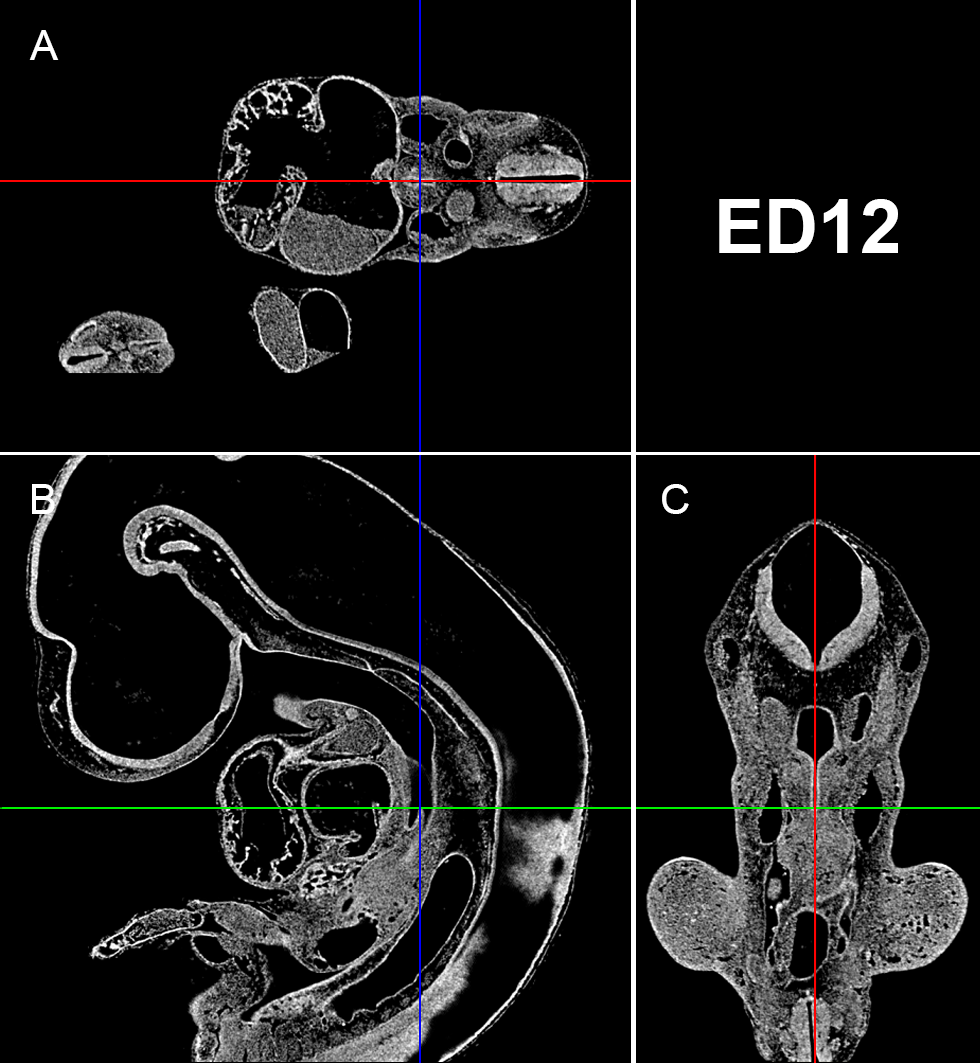

Supplement: Supplementary file 9 [file Image7.TIF]

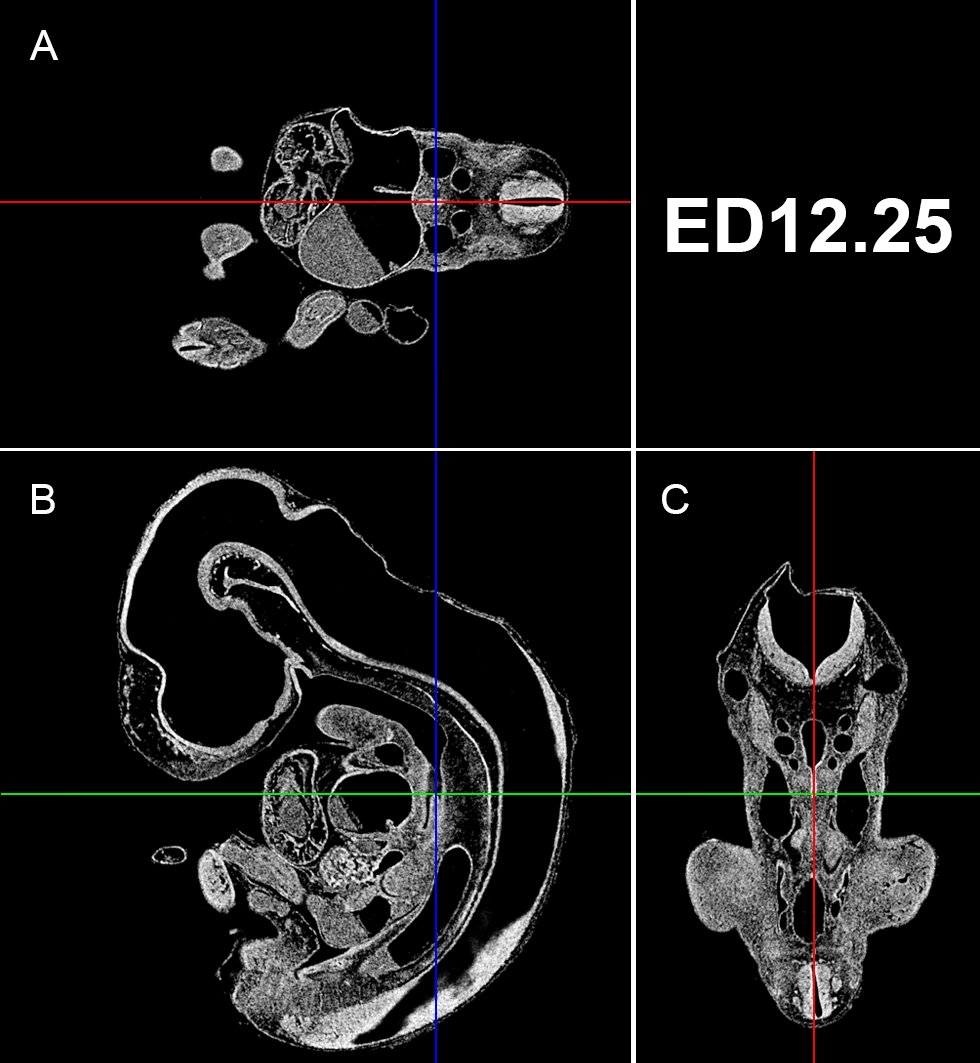

Supplement: Supplementary file 12 [file Image8.TIF]

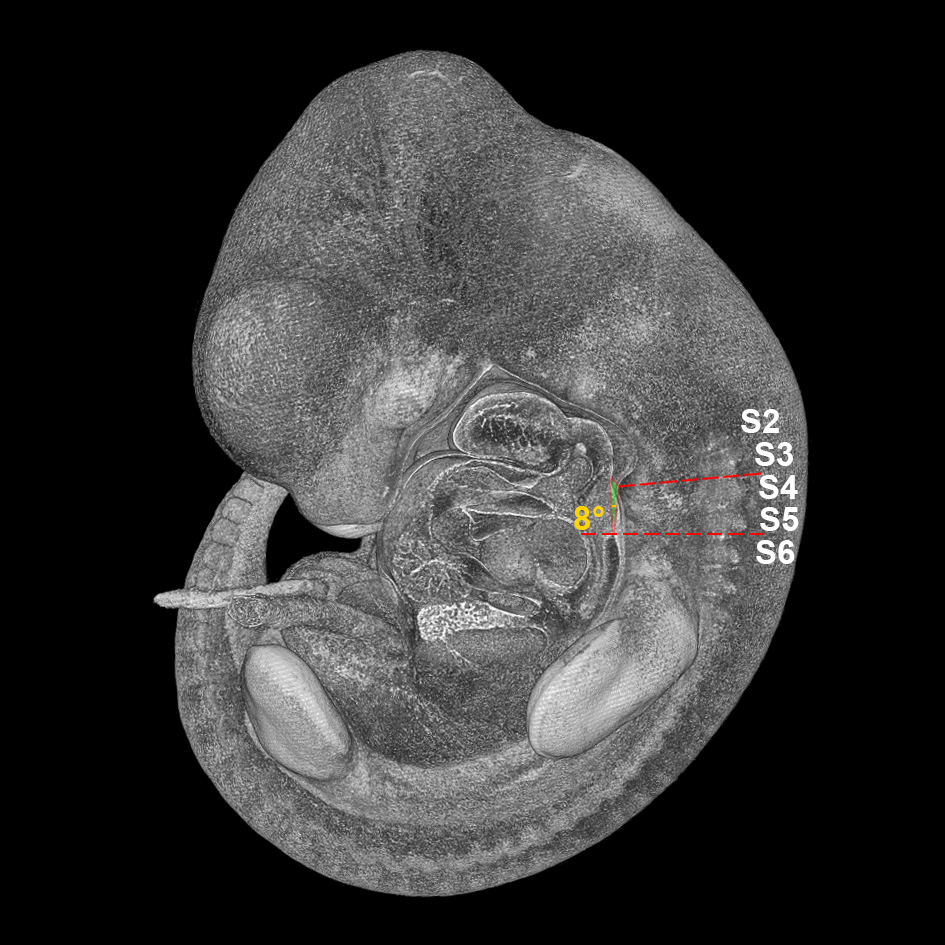

Supplement: Supplementary file 13 [file Image5.TIF]
